# Wild Bitter Melon Leaf Extract Inhibits *Porphyromonas gingivalis*-Induced Inflammation: Identification of Active Compounds through Bioassay-Guided Isolation

**DOI:** 10.3390/molecules21040454

**Published:** 2016-04-06

**Authors:** Tzung-Hsun Tsai, Wen-Cheng Huang, How-Ting Ying, Yueh-Hsiung Kuo, Chien-Chang Shen, Yin-Ku Lin, Po-Jung Tsai

**Affiliations:** 1 Department of Dentistry, Keelung Chang-Gung Memorial Hospital, Keelung 204, Taiwan; tts1725@gmail.com; 2 Department of Pediatrics, Taipei Tzu-Chi Hospital, The Buddhist Tzuchi Medical Foundation, New Taipei City 231, Taiwan; tim810481@yahoo.com.tw; 3 Department of Human Development and Family Studies, National Taiwan Normal University, Taipei 106, Taiwan; rhutuyghk11@yahoo.com.tw; 4 Department of Chinese Pharmaceutical Sciences and Chinese Medicine Resources, China Medical University, Taichung 404, Taiwan; kuoyh@mail.cmu.edu.tw; 5 Department of Biotechnology, Asia University, Taichung 413, Taiwan; 6 National Research Institute of Chinese Medicine, Ministry of Health and Welfare, Peitou, Taipei 112, Taiwan; ccshen@nricm.edu.tw; 7 Department of Chinese Internal Medicine, Keelung Chang-Gung Memorial Hospital, Keelung 204, Taiwan; lin1266@cgmh.org.tw

**Keywords:** *Porphyromonas gingivalis*, wild bitter melon, cucurbitane triterpenoid, anti-inflammation

## Abstract

*Porphyromonas gingivalis* has been identified as one of the major periodontal pathogens. Activity-directed fractionation and purification processes were employed to identify the anti-inflammatory active compounds using heat-killed *P. gingivalis*-stimulated human monocytic THP-1 cells *in vitro*. Five major fractions were collected from the ethanol/ethyl acetate extract of wild bitter melon (*Momordica charantia* Linn. var. *abbreviata* Ser.) leaves and evaluated for their anti-inflammatory activity against *P. gingivalis*. Among the test fractions, Fraction 5 effectively decreased heat-killed *P. gingivalis*-induced interleukin (IL)-8 and was subjected to separation and purification by using chromatographic techniques. Two cucurbitane triterpenoids were isolated from the active fraction and identified as 5β,19-epoxycucurbita-6,23-diene-3β,19,25-triol (**1**) and 3β,7β,25-trihydroxycucurbita-5,23-dien-19-al (**2**) by comparing spectral data. Treatments of both compounds *in vitro* potently suppressed *P. gingivalis*-induced IL-8, IL-6, and IL-1β levels and the activation of mitogen-activated protein kinase (MAPK) in THP-1 cells. Both compounds effectively inhibited the mRNA levels of IL-6, tumor necrosis factor (TNF)-α, and cyclooxygenase (COX)-2 in *P. gingivalis*-stimulated gingival tissue of mice. These findings imply that 5β,19-epoxycucurbita-6,23-diene-3β,19,25-triol and 3β,7β,25-trihydroxycucurbita-5,23-dien-19-al could be used for the development of novel therapeutic approaches against *P. gingivalis* infections.

## 1. Introduction

Periodontitis is a chronic inflammatory disease that compromises the integrity of the tooth-supporting tissues, which include the gingiva, periodontal ligament, and alveolar bone, and are collectively known as the periodontium. *Porphyromonas gingivalis* is a Gram-negative oral anaerobe that has been considered as one of the causative agents of periodontitis [1]. *P. gingivalis* expresses several microbe-associated molecular patterns (MAMPs), such as lipopolysaccharide (LPS) and fimbriae, which are recognized by toll-like receptors (TLR) and lead to the production of pro-inflammatory cytokines [2,3]. Exposure to *P. gingivalis* can trigger the production of pro-inflammatory mediators, such as interleukin (IL)-8, IL-6, IL-1β, and tumor necrosis factor (TNF)-α, in various cell types, including human gingival fibroblasts [4], periodontal ligament stem cells [5], macrophages, and peripheral CD4^+^ T helper cells [6]. These pro-inflammatory mediators may affect the activities of leukocytes, osteoblasts, and osteoclasts and promote the tissue remodeling process systemically and locally [7]. IL-8 has pro-inflammatory and chemo-attractive properties and plays a significant role in the pathogenesis of periodontitis. Continuous and excessive IL-8-mediated chemotactic and activation effects on neutrophils in the inflamed gingiva may contribute to the periodontal tissue destruction [8,9]. IL-6 is a multifunctional cytokine synthesized in response to stimuli such as infection and trauma. This cytokine plays a pro-inflammatory role, increasing its levels and acting on bone resorption in periodontitis [10]. IL-1β, a potent osteotropic cytokine, plays a role in degrading the extracellular matrix in periodontitis [11]. TNF‑α is considered to be a major cytokine involved in the pathogenesis of periodontal disease and enhancing the invasion of *P. gingivalis* [12,13]. Specifically, when compared with healthy individuals, a substantial subset of patients with periodontitis has elevated concentrations of pro-inflammatory cytokines in the gingival tissue [14] and serum [15]. Additionally, the inflammatory mediators that are produced locally in the periodontium could enter the systemic circulation and stimulate acute-phase responses [1]. Therefore, blocking the activity of pro-inflammatory cytokines may be a promising therapeutic modality for periodontitis.

Wild bitter melon (WBM; *Momordica charantia* L. var. *abbreviata* Seringe) is a wild variety of bitter melon (*Momordica charantia*). Leaf and vine extracts of *M. charantia* have been demonstrated to possess broad-spectrum antimicrobial and potent antioxidant actions [16,17], and anti-inflammatory activity against carrageenan-induced rat paw edema [18]. WBM leaf extract possesses significant antioxidant, cyto-protective and anti-melanogenic activities [19]. We previously reported that the polyphenol-enriched fraction from methanolic leaf extract of WBM effectively inhibits *Propionibacterium acnes*-induced inflammatory responses *in vivo*. The anti-inflammatory activity of WBM leaf extract may be attributable to its phenolic and triterpenoid components [20]. Especially, both phenolic and triterpenoid compounds possess wide range of biological activities. Hence, this study was undertaken to evaluate the suppressive effect of WBM leaf extract on *P. gingivalis*-induced inflammation and to identify anti-inflammatory compounds through *in vitro* bioassay-guided isolation approach. Furthermore, we investigated the molecular action mechanism of isolated bioactive compounds *in vitro* and their anti-inflammatory effect *in vivo*.

## 2. Results

### 2.1. Effects of Leaf Extract of Wild Bitter Melon and Its Subfractions on P. gingivalis-Induced Cytokines in THP-Cells

**Figure 1:**
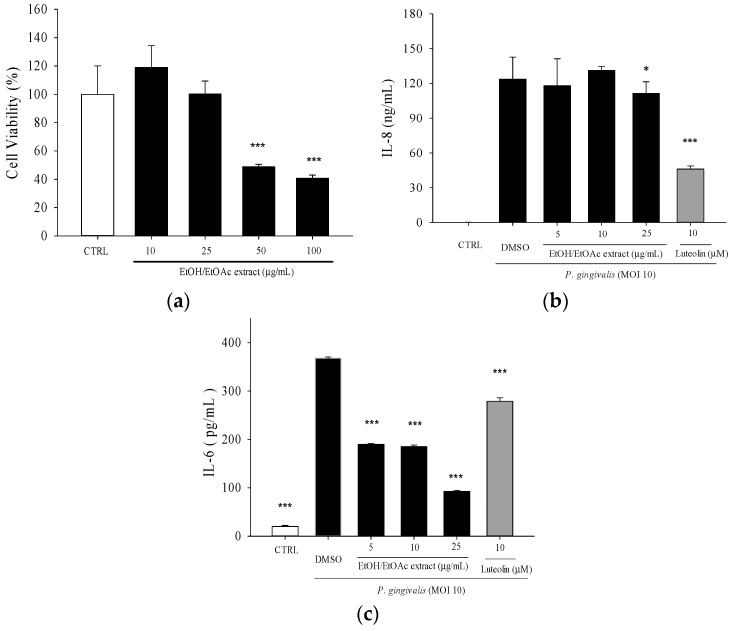
Ethanol/ethyl acetate (EE) extract of WBM leaf inhibited heat-killed *P. gingivalis*-induced cytokine production by THP-1 cells. (**a**) Cytotoxic effect of EE extract on THP-1 cells was determined by MTT assay. *** *p* < 0.001 *vs.* CTRL with 0.1% DMSO alone; (**b**) THP-1 cells were co-incubated with 0.1% DMSO (as vehicle) or the indicated concentrations of EE extract and *P. gingivalis* (M.O.I. = 10) for 24 h. A control experiment with 0.1% DMSO (CTRL) without *P. gingivalis* treatment was conducted in parallel. The culture supernatants were subsequently isolated and analyzed for IL-8 and (**c**) IL-6 concentrations. Luteolin was a positive control. Each column shows the mean ± SD of three independent experiments, each done in quadruplicate. * *p* < 0.05 and *** *p* < 0.001 *vs.* DMSO vehicle (*P. gingivalis* alone).

The ethanol/ethyl acetate (EE) extract of WBM leaf at concentration of 25 µg/mL was not cytotoxic toward THP-1 cells (Figure 1a). Luteolin, up to 20 μM, had no significant cytotoxicity toward THP-1 cells [20]. At the non-cytotoxic concentrations, treatment of ethanol/ethyl acetate extract significantly decreased *P. gingivalis*-induced IL-8 and IL-6 productions (Figure 1b,c).

**Figure 2:**
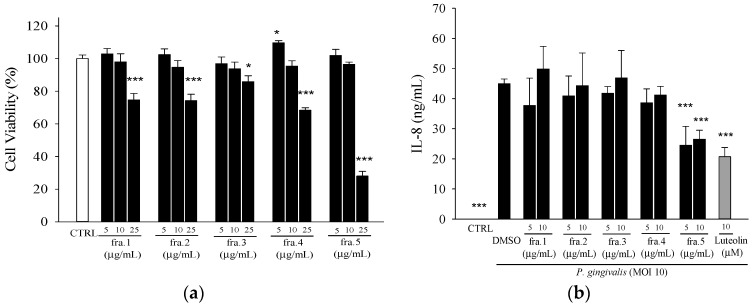
Effects of five sub-fractions from ethanol/ethyl acetate extract of WBM leaf on heat-killed *P. gingivalis*-induced IL-8 production. (**a**) Cell viability was determined by MTT assay. Cells incubated with 0.1% DMSO alone (CTRL), or the indicated concentrations of subfraction for 24 h. * *p* < 0.05 and *** *p* < 0.001 *vs.* CTRL; (**b**) IL-8 level was determined in cells co-incubated with *P. gingivalis* (M.O.I. = 10) and the indicated concentrations of samples for 24 h. Luteolin was a positive control. A control experiment (CTRL) without *P. gingivalis* treatment was conducted in parallel. Each column shows the mean ± SD of three independent experiments, each done in quadruplicate. *** *p* < 0.001 *vs.* DMSO vehicle (*P. gingivalis* alone).

In order to begin the process of identifying the active compounds, the EE extract was divided into five fractions, which were then tested for the inhibitory effect against *P. gingivalis*-induced IL-8 production. Of the five fractions, Fra. 5 significantly inhibited IL-8 levels at concentrations of 5 and 10 µg/mL, which are not cytotoxic toward THP-1 cells (Figure 2).

**Figure 3:**
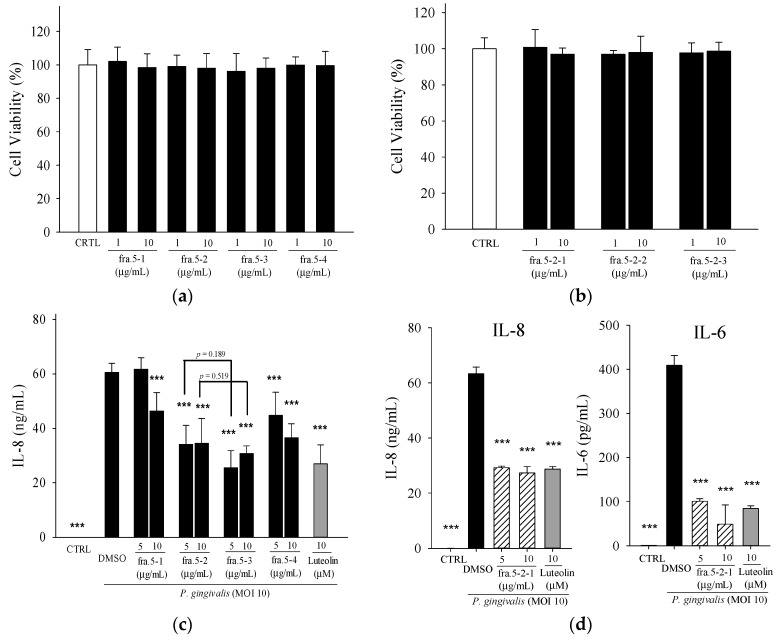
Effects of sub-fractions obtained from the active fraction, Fraction 5, on *P. gingivalis*-induced cytokines in THP cells. (**a**,**b**) Cell viability was determined by MTT assay; (**c**,**d**) IL-8 or IL-6 level was determined in THP-1 cells co-incubated with *P. gingivalis* (M.O.I. = 10) and the indicated concentrations of samples for 24 h. Luteolin was a positive control. A control experiment (CTRL) without *P. gingivalis* treatment was conducted in parallel. Each column shows the mean ± SD of three independent experiments, each done in quadruplicate. *** *p* < 0.001 *vs.* DMSO vehicle (*P. gingivalis* alone).

Fractionation of the active fraction, Fra. 5, yielded four sub-fractions. The IL-8 inhibition assay revealed active four sub-fractions at the concentration of 10 µg/mL, which is not cytotoxic toward THP-1 cells (Figure 3a,b). Fraction 5-2 and 5-3 showed higher inhibition of IL-8 secretion than the other sub-fractions (Figure 3c). Among these four sub-fractions, Fraction 5-2 showed potent inhibitory effect against *P. acnes*-induced inflammation responses [21]; thereby, this fraction was selected for further purification. Three sub-fractions (Fraction 5-2-1, Fraction 5-2-2, and Fraction 5-2-3) were collected from the purification of the compounds in Fraction 5-2. In addition, Fraction 5-2-1 showed the most potent anti-inflammatory activity against *P. acnes* [21]; thereby, this sub-fraction was used to test its inhibitory effect on *P. gingivalis*-induced IL-8 production. Fraction 5-2-1 (5 µg/mL) showed significant inhibition of IL-8 and IL-6 and a similar inhibition as luteolin (10 µM) (Figure 3d). Further purification of Fraction 5-2-1, yielded two pure compounds, 5β,19-epoxycucurbita-6,23-diene-3β,19,25-triol (**1**) and 3β,7β,25-trihydroxycucurbita-5,23-dien-19-al (**2**), as determined by NMR analyses.

### 2.2. Effects of 5β,19-Epoxycucurbita-6,23-diene-3β,19,25-triol *(**1**)* and 3β,7β,25-Trihydroxycucurbita-5,23-dien-19-al *(**2**)* on P. gingivalis-Induced Cytokines in THP-Cells

**Figure 4:**
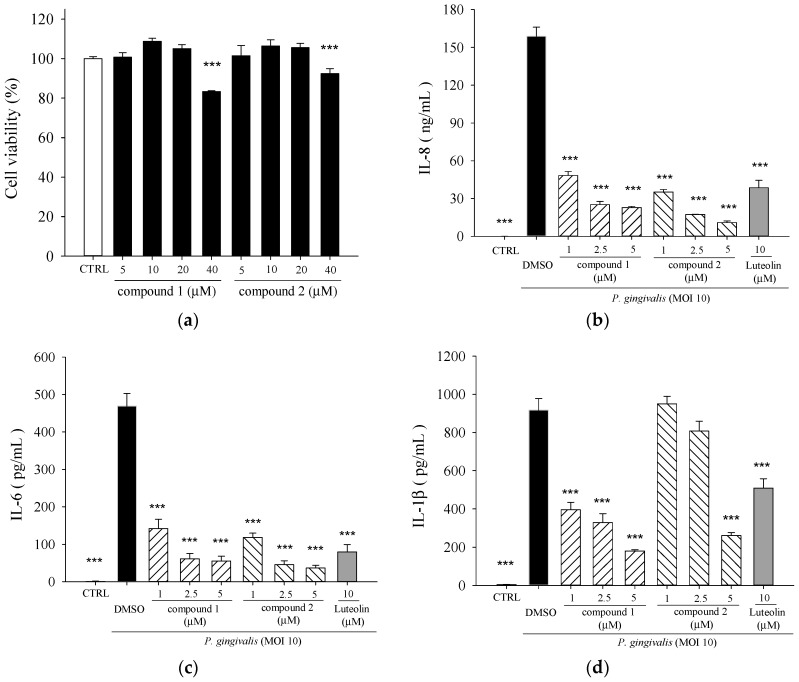
Effects of 5β,19-epoxycucurbita-6,23-diene-3β,19,25-triol (**1**) and 3β,7β,25-trihydroxycucurbita-5,23-dien-19-al (**2**) on *P. gingivalis*-induced cytokines in THP cells. (**a**) Cell viability was determined by MTT assay. *** *p* < 0.001 *vs.* CTRL; (**b**–**d**) THP-1 cells were treated with the indicated concentrations of both compounds in the presence of *P. gingivalis* for 24 h. A control experiment (CTRL) without *P. gingivalis* treatment was conducted in parallel. The culture supernatants were subsequently isolated and analyzed for cytokine concentrations. Each column shows the mean ± SD of three independent experiments, each done in quadruplicate. *** *p* < 0.001 *vs.* DMSO vehicle (*P. gingivalis* alone).

Compounds **1** and **2** were cytotoxic to THP-1 cells when the concentrations applied were higher than 40 μM (Figure 4a). Non-toxic concentrations were used for the subsequent *in vitro* experiments. Both compounds exhibited potent inhibitory activities on *P. gingivalis*-induced IL-8 (Figure 4b), IL-6 (Figure 4c), and IL-1β (Figure 4d) secretions, and were more effective than luteolin. 5β,19-epoxycucurbita-6,23-diene-3β,19,25-triol (**1**) had the highest inhibitory effect on IL-1β production as compared to 3β,7β,25-trihydroxycucurbita-5,23-dien-19-al (**2**) or the positive control, luteolin.

### 2.3. Effects of 5β,19-Epoxycucurbita-6,23-diene-3β,19,25-triol *(**1**)* and 3β,7β,25-Trihydroxycucurbita-5,23-dien-19-al *(**2**)* on MAPK Activation in P. gingivalis-Stimulated THP-1 Cells

**Figure 5:**
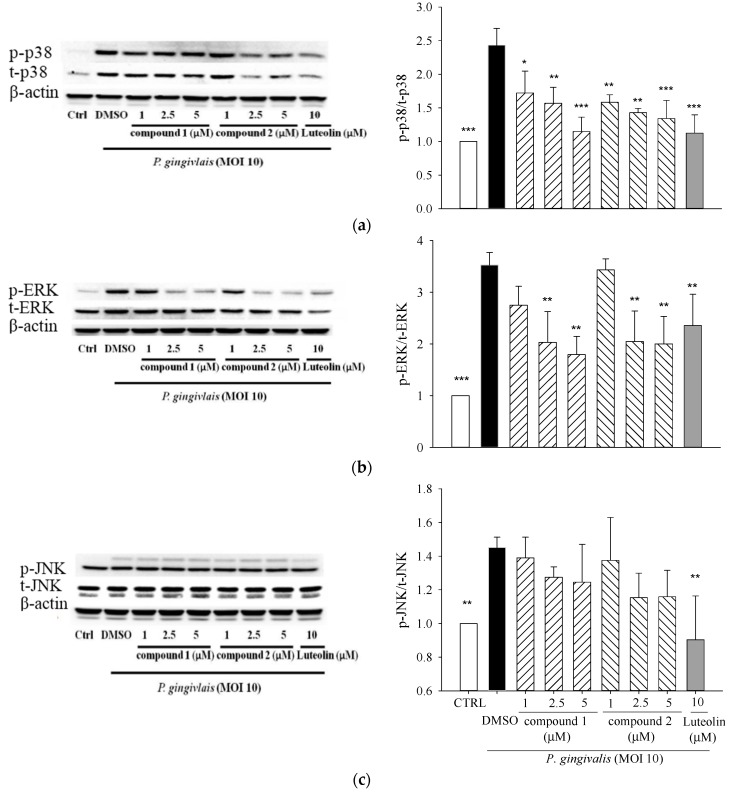
Effects of 5β,19-epoxycucurbita-6,23-diene-3β,19,25-triol (**1**) and 3β,7β,25-trihydroxycucurbita-5,23-dien-19-al (**2**) on the activations of (**a**) p-38 MAPK; (**b**) ERK; and (**c**) JNK proteins in *P. gingivalis*-stimulated THP-1 cells. Each column shows the mean ± SD of three independent experiments. * *p* < 0.05; ** *p* < 0.01; and *** *p* < 0.001 *vs.* DMSO vehicle (*P. gingivalis* alone).

To elucidate the underlying anti-inflammatory mechanism, we evaluated the inflammation-related signaling cascades, MAPK including extracellular signal-related kinase (ERK), p38-mitogen-activated kinase (p38), and c-Jun N-terminal kinase (JNK). The levels of phosphorylated p38, ERK, and JNK were measured by Western blot. Our preliminary study showed that the levels of phosphorylated of MAPKs significantly increased at 15 min, peaked at 30 min, and then declined to control levels at 2 h after *P. gingivalis* stimulation. In the subsequent experiments, the levels of phosphorylated p38, JNK, and ERK were evaluated after 30 min of stimulation. Treatments of compounds **1** and **2** markedly decreased *P. gingivalis*-induced phosphorylation of p38 (Figure 5a) and ERK (Figure 5b) but did not significantly change JNK activation (Figure 5c).

### 2.4. Effect of 5β,19-Epoxycucurbita-6,23-diene-3β,19,25-triol *(**1**)* and 3β,7β,25-Trihydroxycucurbita-5,23-dien-19-al *(**2**)* on mRNA Levels of IL-6, TNF-α, and cyclooxygenase (COX)-2 in P. gingivalis-Stimulated Gingival Tissue of Mice

**Figure 6:**
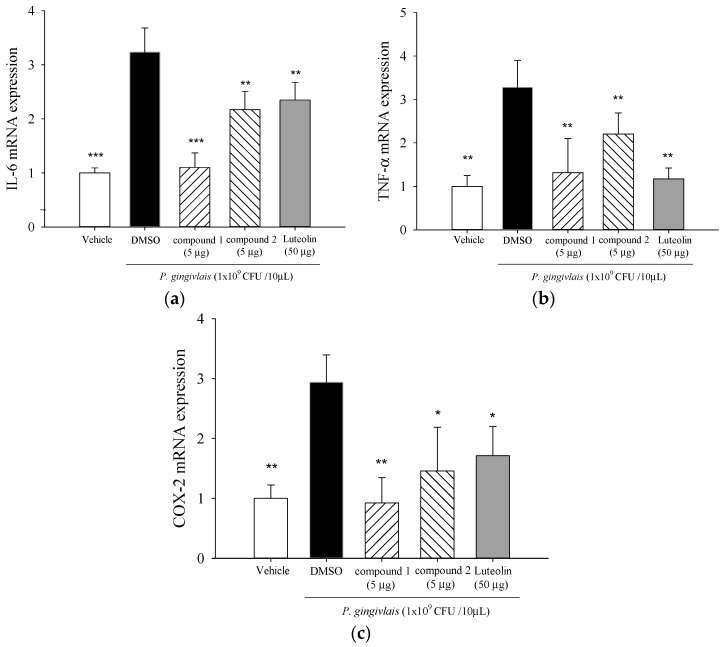
5β,19-epoxycucurbita-6,23-diene-3β,19,25-triol (**1**) and 3β,7β,25-trihydroxycucurbita-5,23-dien-19-al (**2**) suppressed pro-inflammatory cytokine mRNA expressions in *P. gingivalis*-stimulated gingival tissue of mice. Each column shows the mean ± SD (*n* = 5). * *p* < 0.05, ** *p* < 0.01, and *** *p* < 0.001 *vs.* DMSO vehicle (*P. gingivalis* alone).

Pro-inflammatory cytokines (IL-6 and TNF-α) and COX-2 mRNA levels in mice gingival tissues were significantly elevated at day 14 in the *P. gingivalis*-infected group as compared to levels in the un-infected group (Figure 6). Co-injection of 5β,19-epoxycucurbita-6,23-diene-3β,19,25-triol (**1**) or 3β,7β,25-trihydroxycucurbita-5,23-dien-19-al (**2**) resulted in significant reductions of *P. gingivalis*-induced IL-6 (Figure 6a), TNF-α (Figure 6b) and COX-2 (Figure 6c) gene expressions.

## 3. Discussion

Phytotherapeutic agents with antimicrobial and anti-inflammatory properties may be useful for the prevention and treatment of inflammatory periodontal diseases [22,23]. Numerous studies have demonstrated that fruits and leaves of WBM exhibited biological activities associated with anti-inflammation, including those of suppressing nitric oxide (NO) and prostaglandin E2 (PGE_2_) productions in LPS-stimulated Raw 264.7 macrophages [24], reducing IL-8, IL-1β, and TNF-α levels in *P. acnes*-stimulated monocytic THP-1 cells [25], inhibiting infiltrations of neutrophils and IL-1β+ leukocytes in *P. acnes*-inflamed ears of mice [20], and reducing pro-inflammatory cytokine concentrations in the spleen of mice with sepsis [26]. However, the knowledge concerning the active anti-inflammatory ingredients in WBM is still limited. We previously reported that phytol (a diterpene) and lutein (a carotenoid) found in WBM fruits inhibited *P. acnes*-induced pro-inflammatory cytokine releases in THP-1 cells [25]. As part of our continuing efforts directed toward the discovery of structurally interesting, and biologically active, anti-inflammatory agents from WBM, the activity-directed fractionation was used to identify bioactive compounds in this study.

The pathogenic factors of *P. gingivalis* including fimbriae, hemagglutinin, capsule, LPS, outer membrane vesicles, organic metabolites, and various enzymes could contribute to the induction of chronic periodontitis in diverse ways [27]. Our preliminary work revealed that IL-8 concentration increased up to four-fold after the 24-h challenge with heat-killed *P. gingivalis* (at the multiplicity of infection (M.O.I.) = 10, 50, or 100) as compared to the unchallenged negative control. Therefore, treatment of THP-1 cells with heat-killed *P. gingivalis* (M.O.I. = 10) was used as an *in vitro* model to explore the bioactive components from WBM leaf. Our data demonstrated WBM leaf extract and its sub-fractions effectively inhibited *P. gingivalis*-induced pro-inflammatory cytokines (Figure 1, Figure 2 and Figure 3).

Bioactive phytochemical constituents in *M. charantia* have the nutritional and medicinal benefits. A variety of cucurbitane-type triterpenoids has been identified from the fruits, seeds, roots, vines and leaves of *M. charantia* [28]. More than twenty cucurbitane triterpenoids have been reported from the stems and leaves of WBM originated in Taiwan, these cucurbitane triterpenoids possess the *in vitro* modulating estrogen receptors [29], cytoprotective [30], anti-tumor [31] and hypoglycemic activities [32] and, *in vivo*, suppress chemical-induced skin carcinogenesis [33]. 3β,7β,25-trihydroxycucurbita-5,23-dien-19-al (**2**) has been isolated from the fruits of *M. charantia* [34] and stems of WBM [32] and proved to possess anti-diabetic activity. 5β,19-epoxycucurbita-6,23-diene-3β,19,25-triol (**1**) was isolated from stems and leaves of *M. charantia* [35]. However, few have characterized the active components in the WBM extracts responsible for preventing periodontitis. In this study, the total triterpene saponin content of Fraction 5 was 352 mg·g^−1^ by the colorimetric method of Xiang *et al.* [36], using a standard calibration curve of protopanaxadiol. Two cucurbitane triterpenoids, 5β,19-epoxycucurbita-6,23-diene-3β,19,25-triol (**1**) and 3β,7β,25-trihydroxycucurbita-5,23-dien-19-al (**2**), were identified in this active fraction, Fraction 5-2-1. The anti-inflammatory effect of both compounds from WBM leaves against *P. gingivalis* was characterized for the first time. Using the analyses of thin layer chromatography, the Fraction 5-2-1 was found to be enriched in leaf, but not fruit or vine, from various kinds of WBM, including one wild variety and cultivars of Hualien No. 1, Hualien No. 2, Hualien No. 4, #1621, and #5523 [21]. Hence, WBM leaves are good sources of 5β,19-epoxycucurbita-6,23-diene-3β,19,25-triol (**1**) and 3β,7β,25-trihydroxycucurbita-5,23-dien-19-al (**2**). In addition, Fraction 5-3 also showed potent suppressive effect on IL-8 production as compared to Fraction 5-2 (Figure 3c). Fraction 5-3 is worthy to investigate its bioactive components in the further study.

*P. gingivalis* and its components induce NF-κB-containing genes through either TLR2- or TLR7-MyD88-p38 MAPK pathways [37]. *P. gingivalis* induces IL-6 and IL-8 expression through oligomerization domains (NOD) 1/2-mediated NF-κB and ERK1/2 signaling pathways beyond TLRs [38]. MAPKs are intracellular enzymes that are involved in the response of cells to stimuli such as inflammatory cytokines [39].The pro-inflammatory cytokines, such as IL-1 and IL-6, TNFs, have been implicated in the stimulation of osteoclastic resorption in periodontitis [40]. 5β,19-epoxycucurbita-6,23-diene-3β,19,25-triol (**1**) and 3β,7β,25-trihydroxycucurbita-5,23-dien-19-al (**2**) significantly suppressed *P. gingivalis*-induced *in vitro* cytokine productions (Figure 4) and activation of p38 MAPK and ERK proteins in THP-1 cells (Figure 5). It is now accepted that the amplification of initial local host response (lasting approximately 21 days) results in the propagation of the inflammation and leads to the destruction of soft and mineralized periodontal tissues [41]. Elevated COX-2 protein expression is observed in gingival biopsies from patients with chronic periodontitis and gingivitis, and is inversely correlated with the amount of connective tissue in the lamina propria [42]. COX-2 converts arachidonic acid into PGE_2_ which play an important role in periodontitis by mediating pro-inflammatory reactions and the resorption of alveolar bone in the periodontal tissues [43]. Our data revealed that treatment of 5β,19-epoxycucurbita-6,23-diene-3β,19,25-triol (**1**) and 3β,7β,25-trihydroxycucurbita-5,23-dien-19-al (**2**) significantly inhibited *in vivo* mRNA levels of IL-6, TNF-α and COX-2 in gingival tissue of mice after 14 days of *P. gingivalis* stimulation (Figure 6).

In summary, our data suggested that the active sub-fraction of WBM leaf extract enriched in 5β,19-epoxycucurbita-6,23-diene-3β,19,25-triol and 3β,7β,25-trihydroxycucurbita-5,23-dien-19-al could abrogate *P. gingivalis*-induced inflammation and prevent periodontal disease progression, through interference with the productions of inflammatory mediators. Further investigation is required for a complete understanding of the molecular mechanism underlying the anti-inflammatory actions of cucurbitane triterpenoids against *P. gingivalis*.

## 4. Materials and Methods

### 4.1. Plant Material

The fresh aerial parts of WBM (Hualien No. 1) were kindly provided by Researcher Jong-Ho Chyuan (Hualien District Agricultural Research and Extension Station, Hualien, Taiwan). WBM leaves were collected and then a voucher specimen was deposited in the Department of Human Development and Family Studies, National Taiwan Normal University. The voucher specimen of the plant was authenticated by Dr. Po-Jung Tsai, Professor, National Taiwan Normal University, Taipei, Taiwan. After cleaning with water, the WBM leaves were air-dried and ground using a blender. Powdered WBM leaves were stored in the dark at −20 °C until used.

### 4.2. Isolation and Determination of the Active Compounds

**Figure 7:**
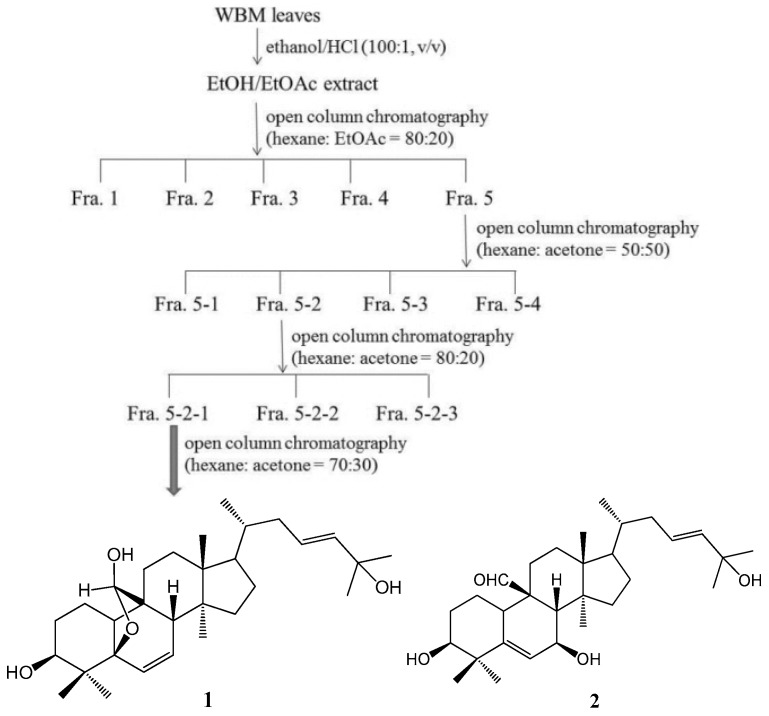
Cucurbitane triterpenoids were isolated and identified on the basis of activity-guided purification procedures. Activity-guided purification procedures were performed with open column chromatography for leaf extract of wild bitter melon. ^1^H nuclear magnetic resonance (NMR) was performed for molecular characterization of the final isolated product that had the most desired biological activities (Fraction 5-2-1). 5β,19-epoxycucurbita-6,23-diene-3β,19,25-triol (**1**) and 3β,7β,25-trihydroxycucurbita-5,23-dien-19-al (**2**) were finally identified.

The WBM leaf extract was prepared using a procedure described by Huang *et al.* [20], with some modification. As shown in Figure 7, dried and powdered WBM leaves (300 g) were extracted twice with 3 L of ethanol/HCl (100:1, *v*/*v*) at room temperature on a rotary shaker at 200 rpm in the dark for 24 h. The blended mixture was then centrifuged at 5000 *g*. The supernatant obtained was filtered, and then evaporated to dryness under reduced pressure (45–50 °C). The residue was re-dissolved in 25 mL of water/ethanol (80:20, *v*/*v*) and extracted four times with 25 mL of ethyl acetate in a separatory funnel. The organic fractions were combined, dried for 30–40 min with anhydrous sodium sulfate, and filtered through a Whatman-40 filter, and evaporated to dryness under vacuum (45–50 °C) to obtain the ethanol/ethyl acetate (EE) extract. A yield of EE extract was 12.2% of the original weight. The EE extract was stored in the dark at −20 °C. Next, the EE extract (120 mg) was dissolved in ethyl acetate and then subjected to chromatographic separation on the open column of silica gel (Silicycle 230−400 mesh) in a small scale pretest using hexane: ethyl acetate (80:20) as eluent, and fractions of 5 mL were collected. The collected fractions were based on their TLC patterns as follow: Fraction 1 (7 mg), Fraction 2 (2 mg), Fraction 3 (5 mg), Fraction 4 (8 mg), and Fraction 5 (50 mg). All extracts were stored in the refrigerator before use. All of the Fraction 5 (25.7 g) were further chromatographed on a silica gel column (35 mm × 45 cm), eluted with hexane: acetone (80:20) to give four fractions (about 1.6 g, 3.9 g, 1.5 g, and 17 g, respectively). Fraction 5-2 (3.9 g) was subjected to the open column of silica gel using *n*-hexane–acetone (50:50) as eluent and to give three fractions. Fraction 5-2-1 (2.9 g) was subjected to the silica gel column eluted with *n*-hexane-acetone (70:30) and to yield 5β,19-epoxycucurbita-6,23-diene-3β,19,25-triol (**1**; 130 mg) and 3β,7β,25-trihydroxycucurbita-5,23-dien-19-al (**2**; 390 mg). Nuclear magnetic resonance (^1^H-NMR and ^13^C-NMR) techniques were used for the structure elucidation of the compounds. NMR spectra were recorded on a Bruker spectrometer (600 MHz for ^1^H-NMR and 150 MHz for ^13^C-NMR) instrument, and using CDCl_3_ as solvent. The isolates were identified as 5β,19-epoxycucurbita-6,23-diene-3β,19,25-triol (**1**), and 3β,7β,25-trihydroxycucurbita-5,23-dien-19-al (**2**) by comparison of their spectroscopic data with those of published in the related references [44,45]. The ^1^H-NMR and ^13^C-NMR, DEPT, COSY, HSQCAD, and HMBCAD spectral data of the two pure compounds obtained in this investigation are available as Supplementary Figures.

### 4.3. Preparation of Heat-Inactivated Porphyromonas Gingivalis

The strain of *Porphyromonas gingivalis* (BCRC14417) was obtained from the Bioresource Collection and Research Center (Hsinchu, Taiwan). Fresh *P. gingivalis* cells from blood agar plates supplemented with hemin (5 μg/mL; Sigma-Aldrich, St Louis, MO, USA) and menadione (1 μg/mL; Sigma-Aldrich) were inoculated into 5 mL of trypticase soy broth (TSB, Difco, Detroit, MI, USA) supplemented with 2.5% yeast extract, hemin, and menadione. The cultures were incubated anaerobically in an N_2_-H_2_-CO_2_ (80:10:10) atmosphere at 37 °C by using a Concept 400 Anaerobic Workstations (Ruskinn, Sanford, ME, USA). Organisms were harvested by centrifugation, washed with phosphate-buffered saline (PBS), and re-suspended in PBS. The numbers of bacteria were determined with a spectrophotometer (at an optical density at 600 nm) based on a standard curve established by colony formation on bacterial plates. To prepare heat-killed *P. gingivalis* cells, bacterial suspensions in PBS were heated at 80 °C for 30 min, washed with PBS, and re-suspended in RPMI 1640 medium (Gibco, Carlsbad, CA, USA).

### 4.4. Cell Culture and Determination of the Viability of THP-1 Cells

A human monocytic leukemia cell line THP-1 that behaves like monocytes and after differentiation also behaves like monocyte-derived macrophages. Treatment of resting THP-1 cells with lipopolysaccharide extracts from *P. gingivalis* causes significant increases in pro-inflammatory cytokines, such as IL-8, IL-6, IL-1β, and TNF-α [46,47]. Hence, the activity-directed fractionation and purification processes were employed to identify the bioactive constituents of WBM leaf extract using heat-killed *P. gingivalis*-stimulated THP-1 cells in this study.

The THP-1 cell line (BCRC 60430) was obtained from the Bioresource Collection and Research Center and maintained in RPMI 1640 (Gibco) supplemented with 10% heat-inactivated fetal bovine serum (FBS, Gibco), penicillin (100 U/mL), and streptomycin (100 μg/mL) at 37 °C in a humidified atmosphere with 5% CO_2_. A suspension of THP-1 cells (1 × 10^6^ cells/mL) was cultured in 96-well culture plates with treatment of various concentrations of tested samples for 24 h at 37 °C in a humidified atmosphere of 5% CO_2_. Whole cell suspension was taken from each well and centrifuged 4 min at 600 *g*. Supernatant was eliminated, and the pellet was incubated 3 h at 37 °C with 100 μL of MTT reagent (Sigma-Aldrich). Samples were centrifuged 2 min at 4500 *g*, and supernatant was gently removed. Finally, the cell pellets were diluted with 500 μL of isopropanol/HCl, and then 200 μL of each sample was transferred in duplicate in a 96-well plate. The absorbance was measured using a using a Synergy HT multi-detection micro-plate reader (Bio-Tek, Carson, NV, USA) at 540 nm with 690 nm as the reference wavelength.

### 4.5. Measurement of Cytokine Production in Human Monocytic THP-1 Cells

The dried EE extract, sub-fractions and compounds (**1** and **2**) were reconstituted in dimethyl sulfoxide (DMSO) for the subsequent experiments. THP-1 cells (2 × 106 cells/mL) were seeded in 96-well plates with serum-free medium, and were stimulated with heat-killed *P. gingivalis* (M.O.I. = 10) alone or in combination with different concentrations of tested samples or luteolin for a 24-h incubation. Cell-free supernatants were collected, and concentrations of IL-6, IL-8, and IL-1β were analyzed with respective enzyme immunoassay kits (Invitrogen, Carlsbad, CA, USA). Luteolin has also been reported to block *P. gingivalis* LPS-mediated COX-2 expression by inhibiting the activation of MAPK phosphorylation in human gingival fibroblasts [48] and, thereby, was used in this study as a positive control.

### 4.6. Detection of MAPK Phosphorylation by Western Blot Analysis

Human monocytic THP-1 cells were seeded at 2 × 10^6^ cells/mL in 6-cm dishes and were stimulated with heat-killed *P. gingivalis* (M.O.I. = 10) co-incubated with various concentrations of tested samples. Cells were harvested and washed with PBS, after 30 min of treatments for the measurement of MAPK phosphorylation. Whole cell lysates were prepared in a lysis buffer (Cell Signaling, Beverly, MA, USA) containing 10 mM phenylmethylsulfonyl fluoride (PMSF). The cell lysates were sonicated and cleared by centrifugation at 4 °C, 14,000 *g* for 10 min. Protein concentrations were determined by DC protein assay (Bio Rad, Hercules, CA, USA). Aliquots of the lysates (each containing 30 μg of protein) were boiled for 5 min and electrophoresed on a 10% sodium dodecyl sulfate (SDS)-polyacrylamide gel. Following SDS-polyacrylamide gel electrophoresis, proteins were transferred to PVDF membranes. Membranes were blocked by incubation in gelatin-NET buffer at room temperature, and then incubated with 1:1000 dilution of primary antibodies of MAPK, phospho-MAPK (Cell Signaling Technology, Danvers, MA, USA) and anti-β-actin (Sigma-Aldrich), followed by horseradish peroxidase-conjugated secondary antibody according to the manufacturer’s instructions. The immunoreactive proteins were detected using the enhanced ECL chemiluminescence Western Blotting Detection System (ChemeDoc XRS, Bio-Rad, Hercules, CA, USA). Signal strengths were quantified using densitometric program (Image Lab, Bio-Rad).

### 4.7. Induction of Experimental Periodontitis by Injection of P. gingivalis

Six-week-old male C57BL/6 mice were obtained from the National Laboratory Animal Center (Taipei, Taiwan). The mice were housed in groups of five per cage, under standard temperature-controlled conditions with a 12 h/12 h light-dark cycle and free access to food and water throughout the experiments. All animal experiments were conducted in accordance with the Guide for the Care and Use of Laboratory Animals and were approved by the Animal Care Committee of the National Taiwan Normal University. Throughout the period of the study mice were fed with sterile standard solid mice chow diet and sterile water. Periodontitis was induced by an intra-gingival injection of *P. gingivalis* according to the method by de Molon *et al.* [49] with some modifications. After ONE week of adaptation, animals were randomly divided into five groups (*n* = 5). Heat-killed *P. gingivalis* (1 × 10^9^ CFU in PBS) or PBS (as vehicle control) was injected once daily into the mandibular (lower inset) gingival tissues of mice for three days. To study the effects of compounds **1** and **2**, they were respectively administered once daily for three days with co-injection of heat-killed *P. gingivalis* suspensions. After 14 days of bacterial injection, mice were then sacrificed with carbon dioxide asphyxiation. The gingival tissues were excised for the extraction of total RNA for reverse transcription qualitative polymerase chain reaction (RT-qPCR).

### 4.8. RNA Isolation and Quantitative Real-Time Polymerase Chain Reaction (PCR)

Total RNA was isolated with the TRIzol reagent (Invitrogen), according to the manufacturer’s instructions. Complementary DNA was generated from 2 μg of total RNA, with the oligo (dT) primer and 1 μL of reverse transcriptase (Promega, Madison, WI, USA). Primers and probes were selected for the genes: IL-6, TNF-α, and COX-2. GAPDH (glyceraldehyde-3-phosphate dehydrogenase) was used as the housekeeping gene. We used the forward GACTGATGCTGGTGACAA and reverse GTGAAGTGGTATAGACAGGTC primers for IL-6, the forward AACGCTTCTTCCAATG and reverse GGCTCTGTTGAGGTCTAA primers for TNF-α, the forward AAAGAAGGGTTCCCAATTAAAGAT and reverse GCATTGAGAGATGGACTGTTAG primers for COX-2, and the forward ACCACAGTCCATGCCATCAC and reverse CACCACCCTGTTGCTGTAGCC primers for GAPDH. These primer pairs amplified, respectively, a 102 bp fragment of the IL-6 cDNA, a 83 bp fragment of the TNF-α cDNA, a 184 bp fragment of the COX-2 cDNA, and a 89 bp fragment of the GAPDH cDNA. Real-time PCRs were conducted in an iCycler iQ Real-Time detection system (Bio-Rad, Hercules, CA, USA) using iQ^TM^ SYBR Green Supermix (Bio-Rad). Thermal cycling conditions for all assays were initial denaturation at 95 °C for 3 min and 40 cycles at 95 °C for 10 s and 51 °C for 30 s. Melting analysis was performed by denaturing at 95 °C for 1 min and cooling to 51 °C for 1 min followed by heating at the rate of 0.5 °C/s from 51 °C to 95 °C. The relative amounts of the PCR products were analyzed by iQ™ 5 optical system software, version 2.1 (Bio-Rad). All expression levels were normalized using the GAPDH as an internal standard in each sample. Fold expression was defined as the fold increase relative to controls.

### 4.9. Statistical Analysis

All data are presented as means ± SD. Statistical analyses were performed using the SPSS 19.0 statistical package (Chicago, IL, USA). The data were evaluated for statistical significance with the one-way ANOVA followed by least significant difference (LSD) tests. A *p* value of <0.05 was considered statistically significant.

## Supplementary Materials

Supplementary materials can be accessed at: http://www.mdpi.com/1420-3049/21/4/454/s1.
