# Supplementary material for: Wild Bitter Melon Leaf Extract Inhibits Porphyromonas gingivalis-Induced Inflammation: Identification of Active Compounds through Bioassay-Guided Isolation
Source: Molecules. 2016 Apr 6;21(4):454. doi: 10.3390/molecules21040454 (PMC6273076; doi:10.3390/molecules21040454)
Supplement: Supplementary file 1 [file molecules-21-00454-s001.pdf]

Supplementary Materials: Wild Bitter Melon Leaf Extract Inhibits *Porphyromonas gingivalis*-Induced Inflammation: Identification of Active Compounds through Bioassay-Guided Isolation

Tzung-Hsun Tsai, Wen-Cheng Huang, How-Ting Ying, Yueh-Hsiung Kuo, Chien-Chang Shen, Yin-Ku Lin and Po-Jung Tsai

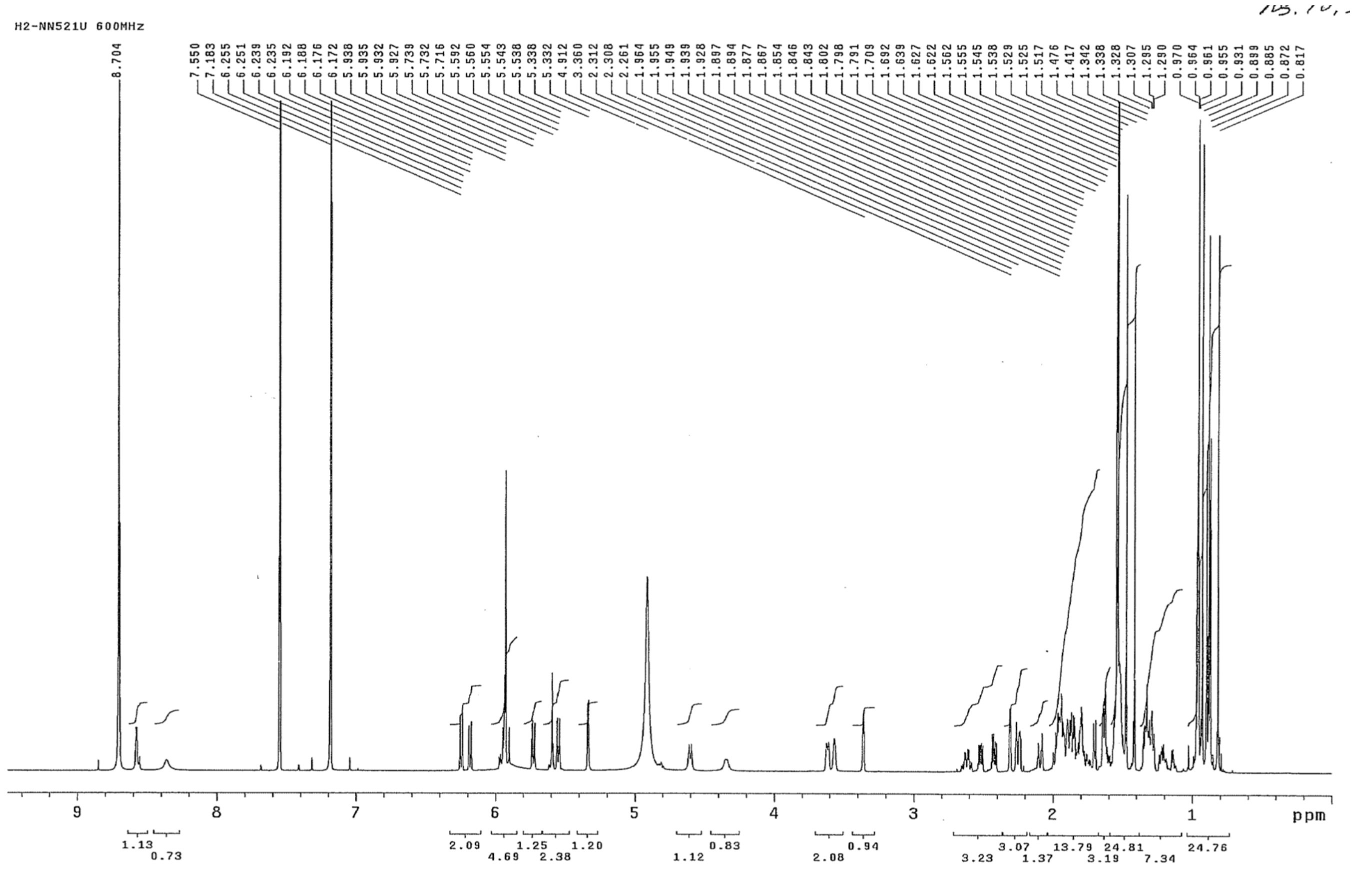

Figure S1. ^{1}H-NMR (600 MHz) of **1**.

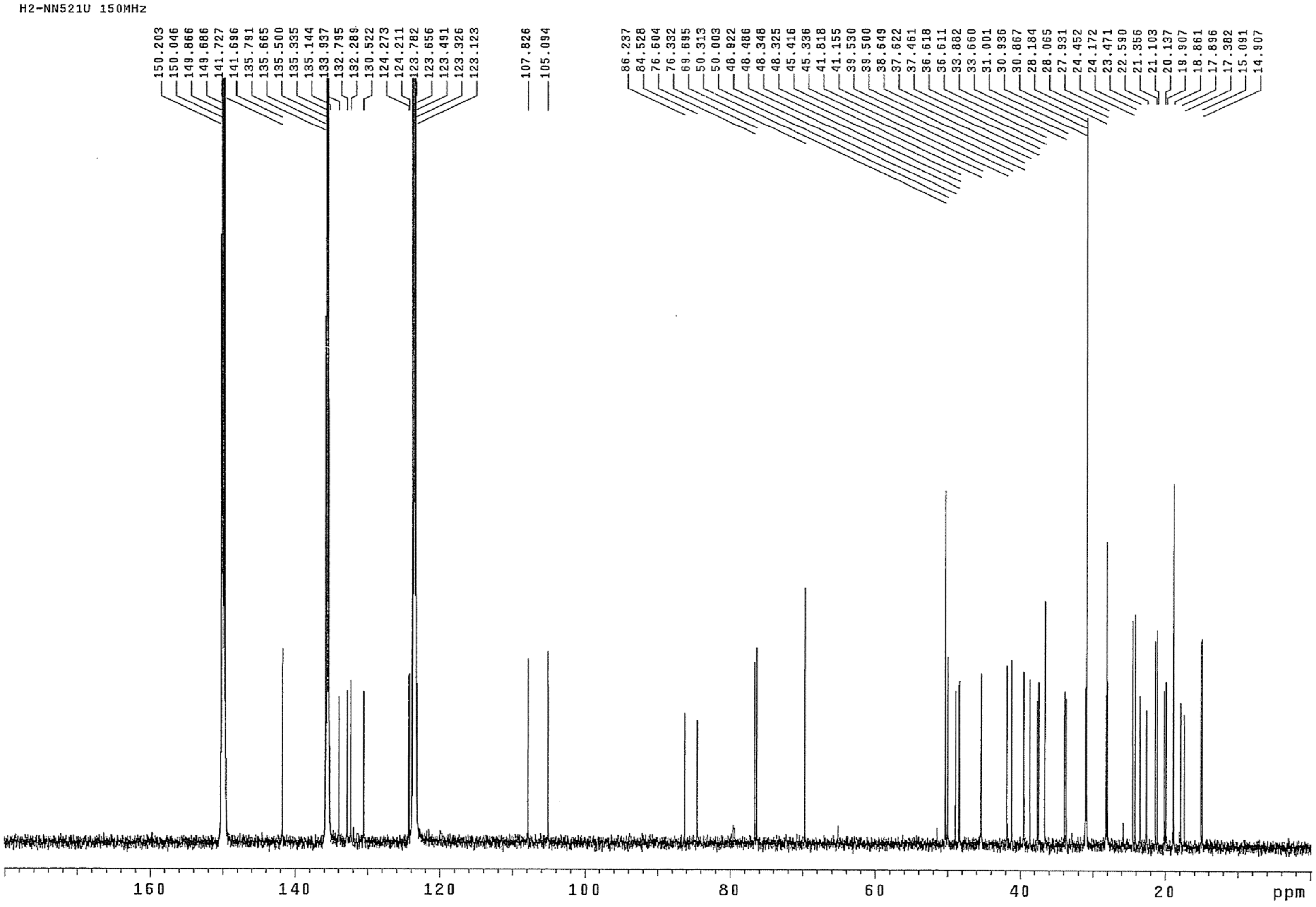

Figure S2. ^{13}C-NMR (150 MHz) of **1**.

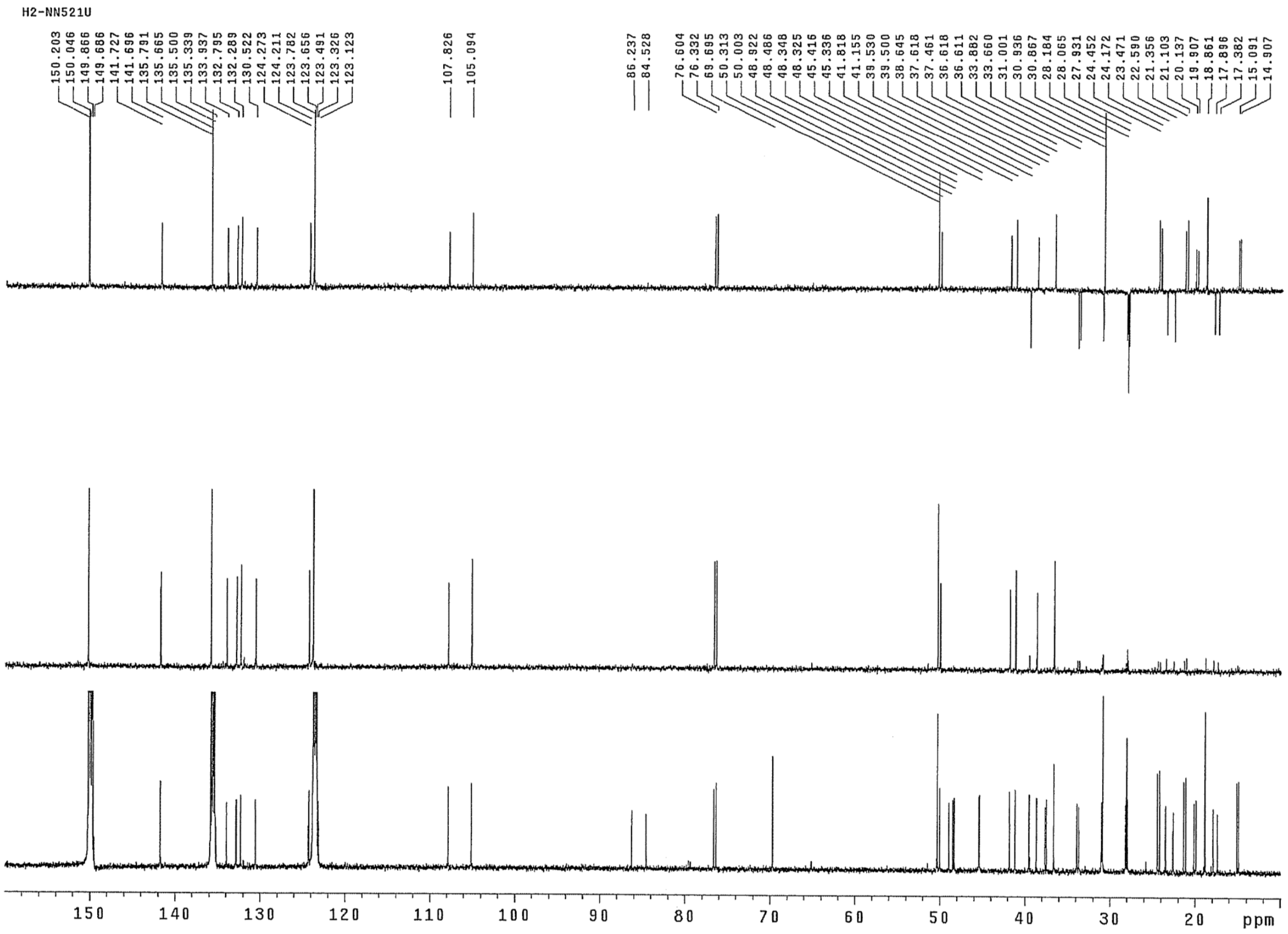

Figure S3. DEPT spectrum of **1**.

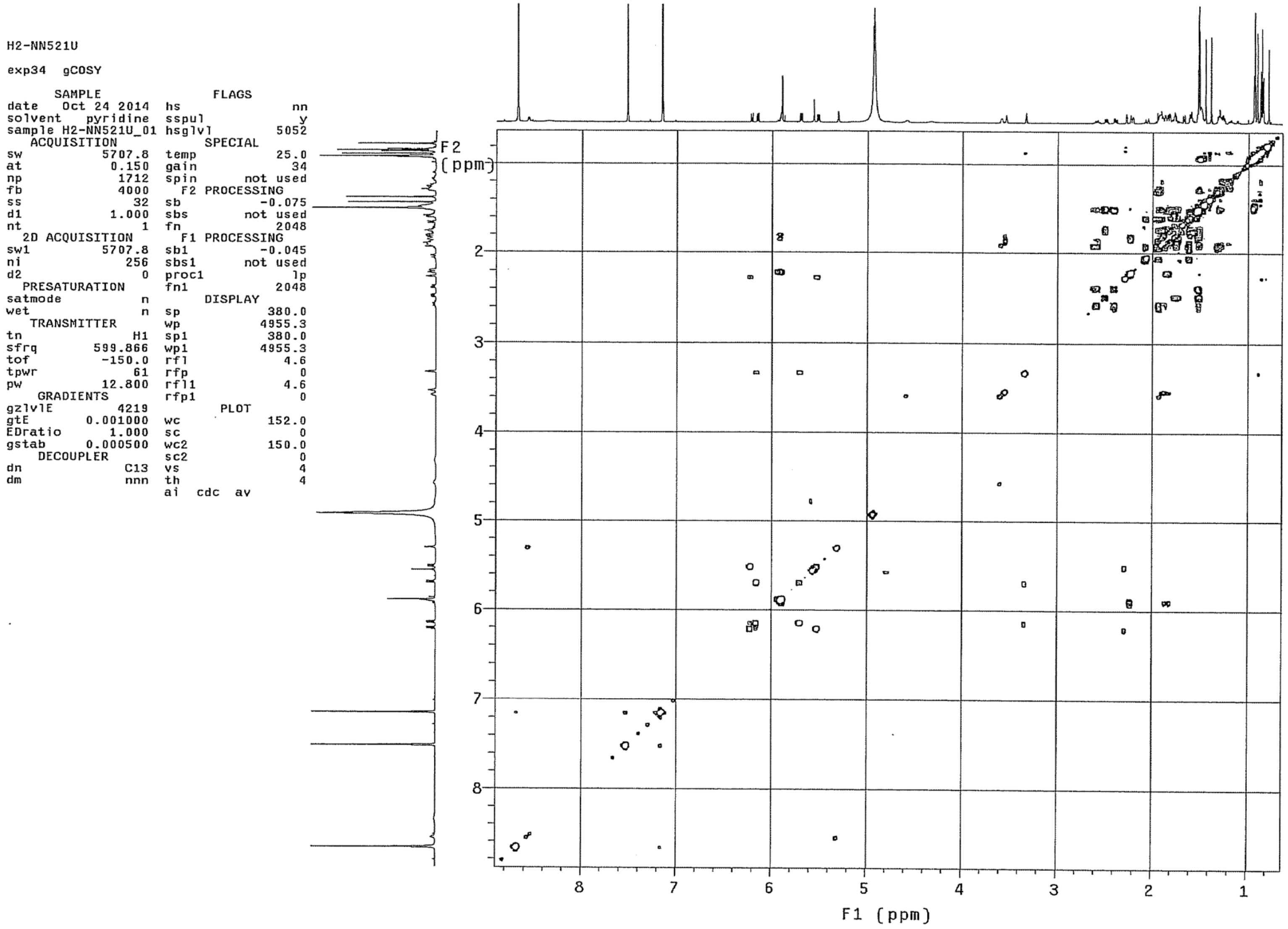

Figure S4. gCOSY of **1**.

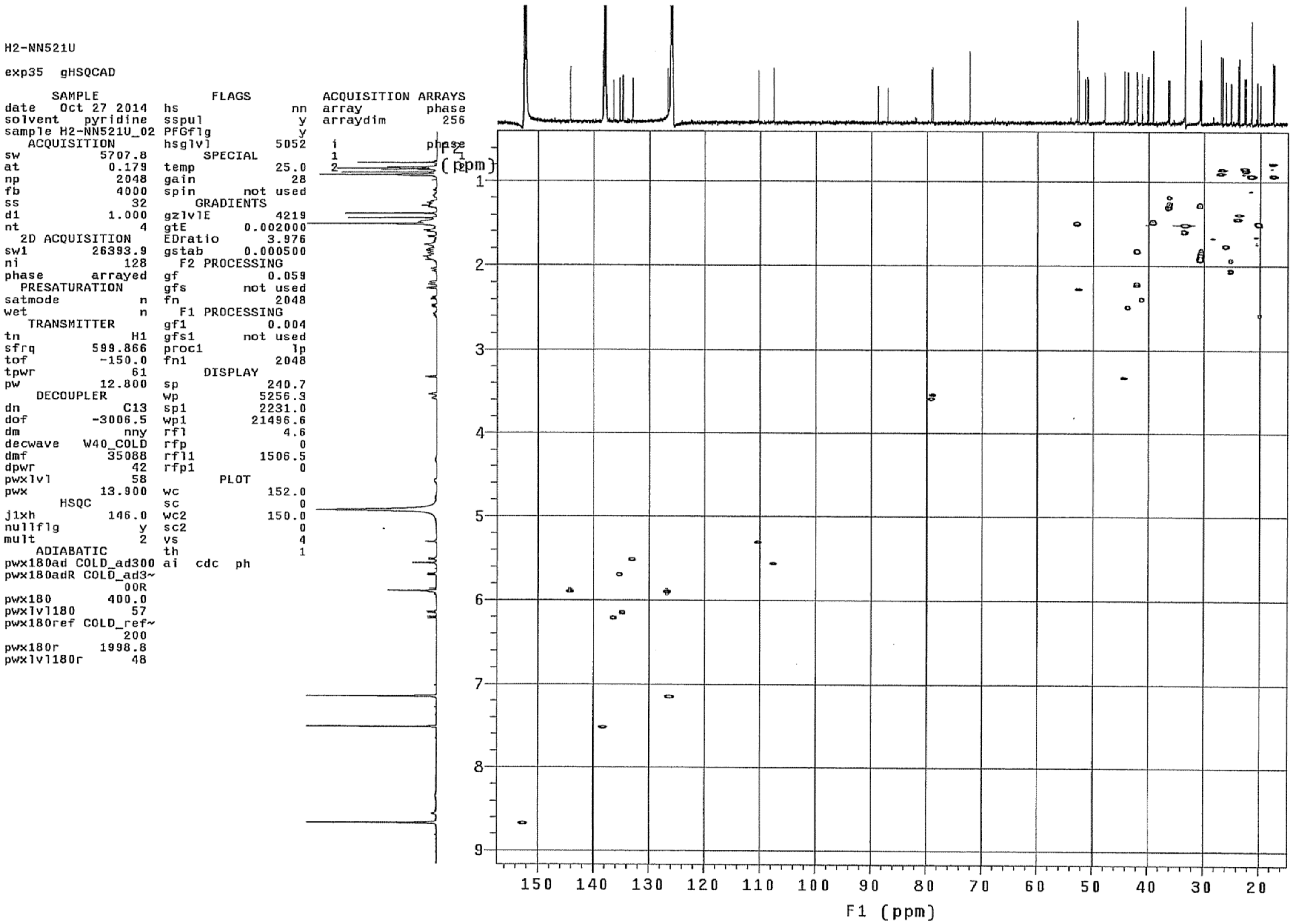

Figure S5. gHSQCAD of **1**.

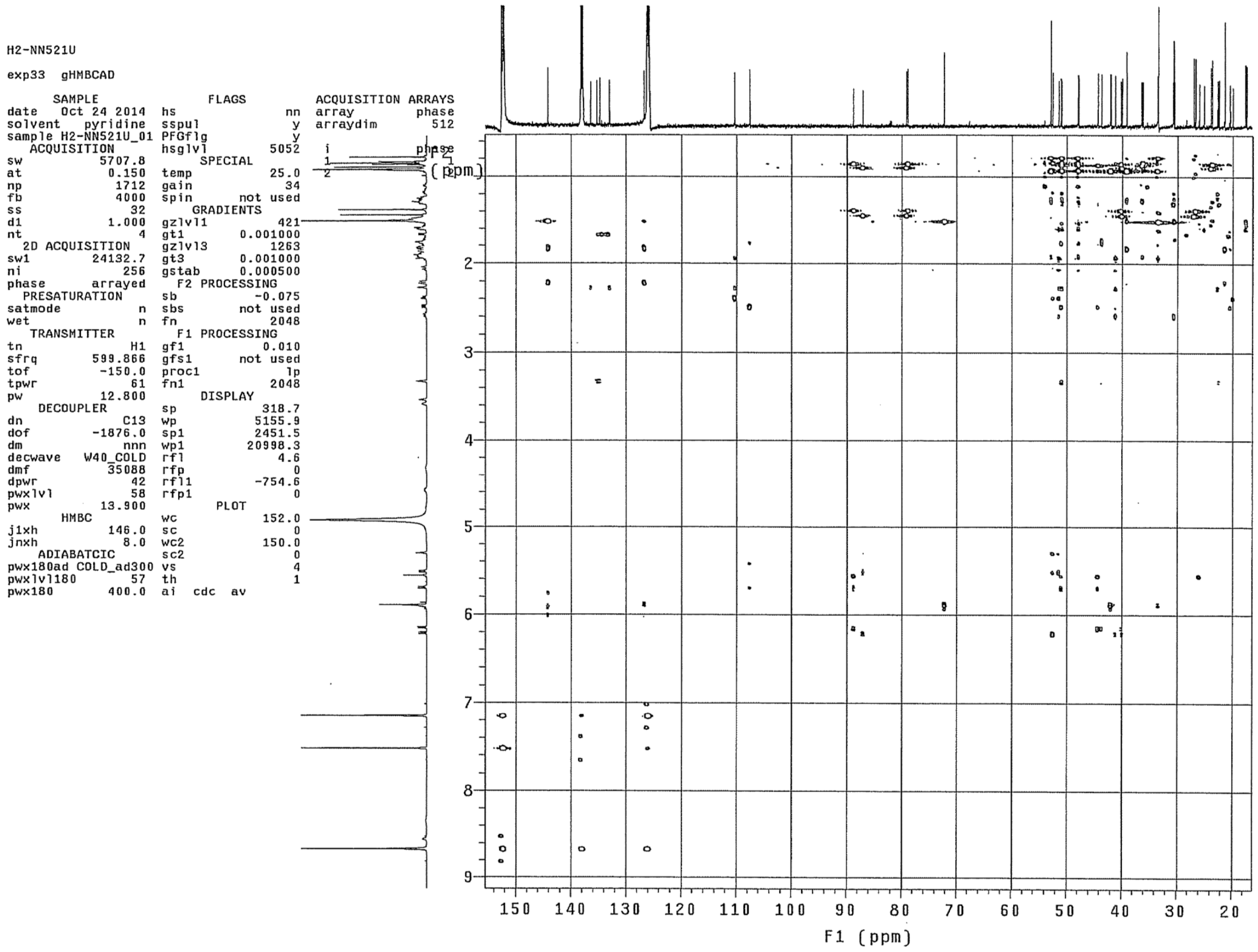

Figure S6. gHMBCAD of **1**.

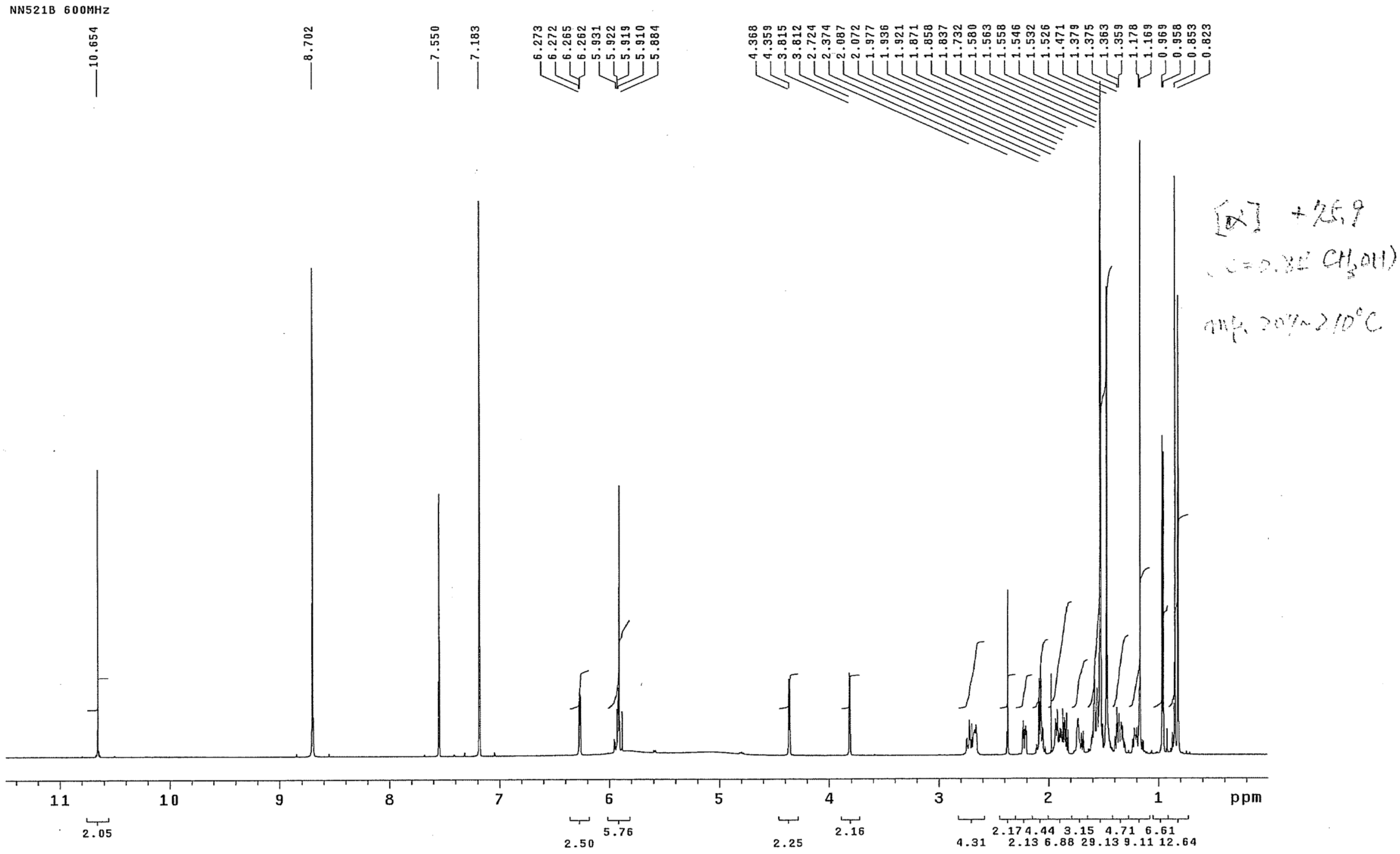

Figure S7. ¹H-NMR (600 MHz) of **2**.

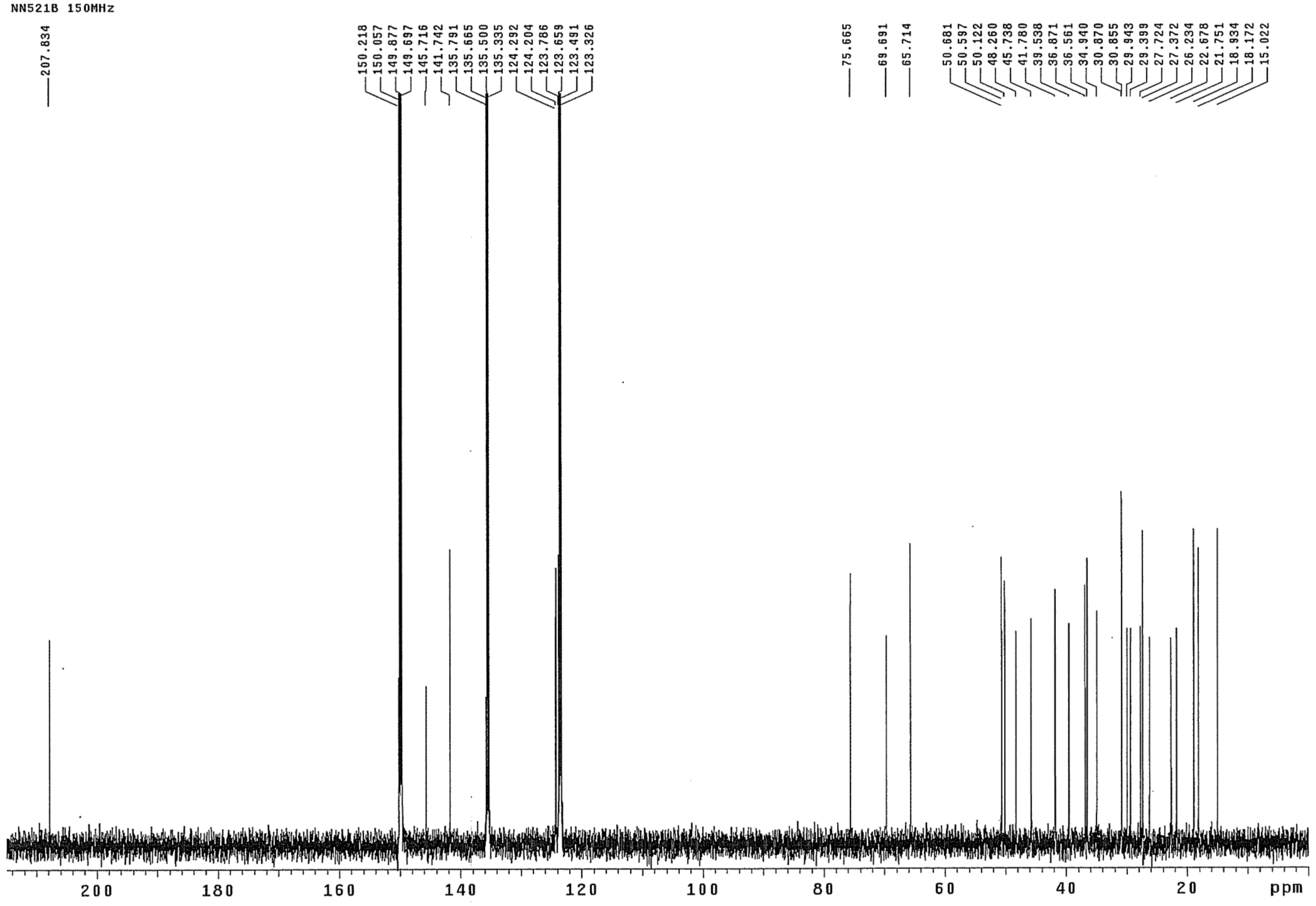

Figure S8. ^{13}C-NMR (150 MHz) of **2**.

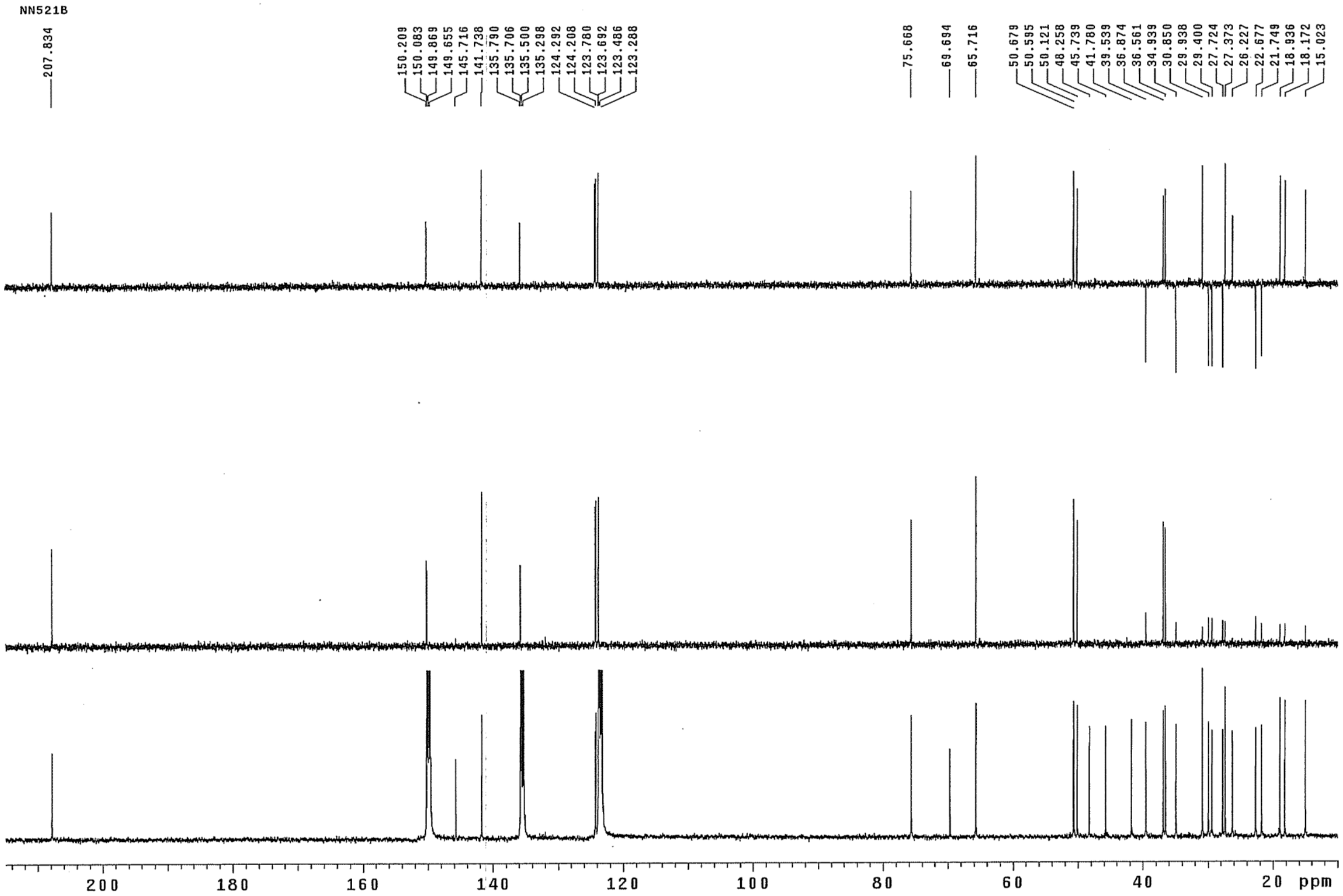

Figure S9. DEPT spectrum of **2**.

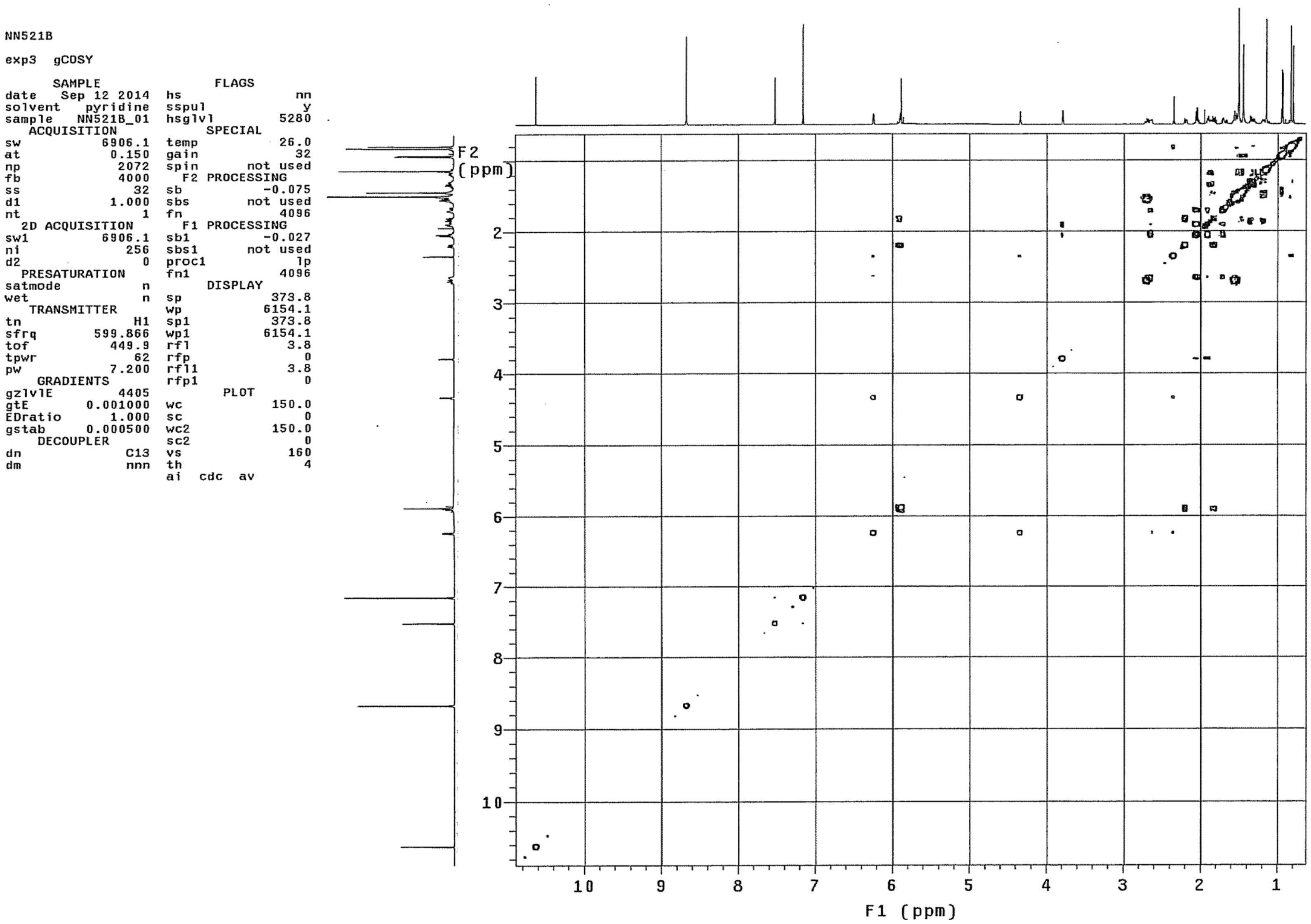

Figure S10. gCOSY of **2**.

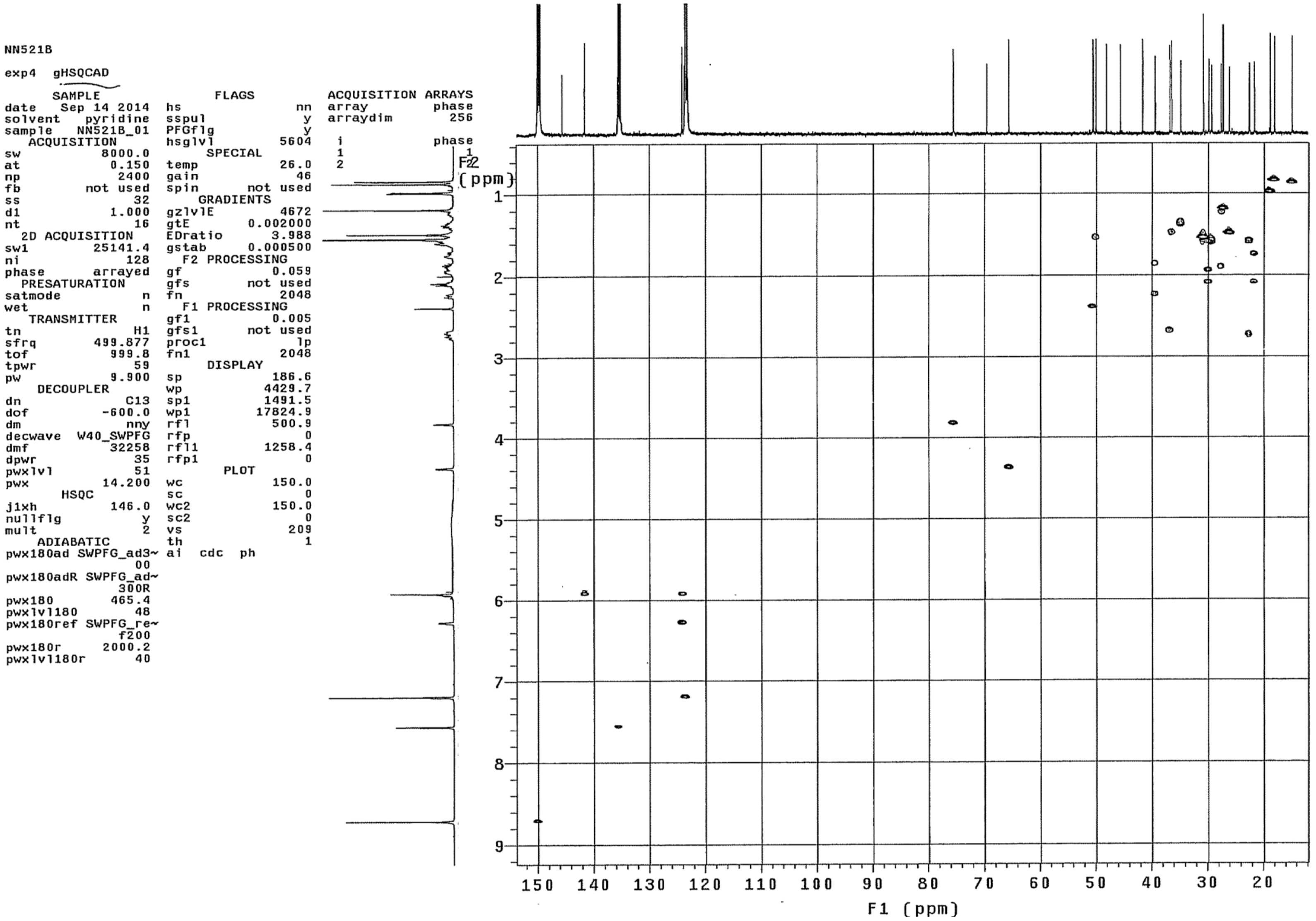

Figure S11. gHSQCAD of compound **2**.

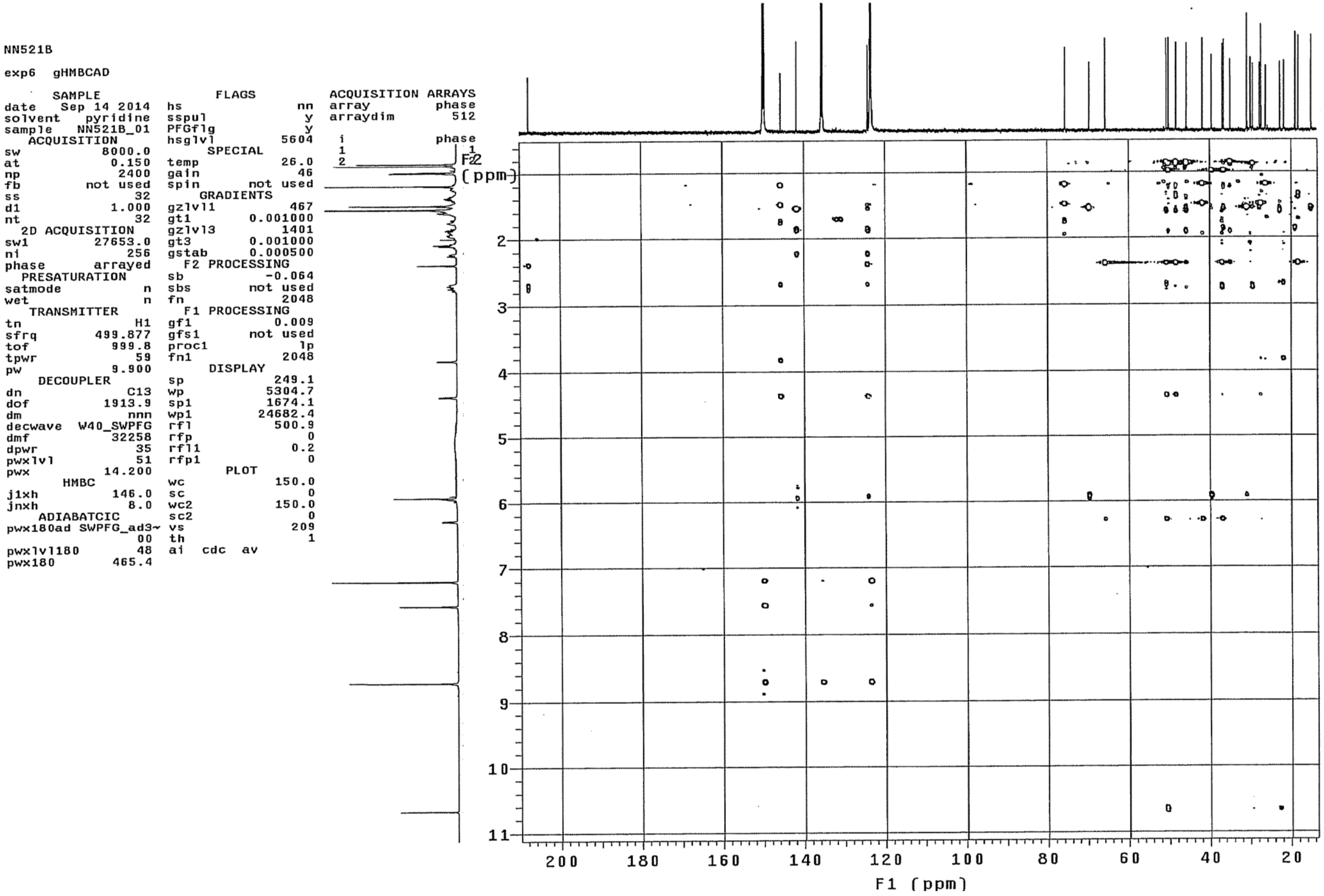

Figure S12. gHMBCAD of **2**.
